# Evaluation of *NAB2-STAT6* Fusion Variants and Other Molecular Alterations as Prognostic Biomarkers in a Case Series of 83 Solitary Fibrous Tumors

**DOI:** 10.3390/cancers13205237

**Published:** 2021-10-19

**Authors:** Carmen Salguero-Aranda, Paula Martínez-Reguera, David Marcilla, Enrique de Álava, Juan Díaz-Martín

**Affiliations:** 1 Instituto de Biomedicina de Sevilla, Department of Pathology, Hospital Univesitario Virgen del Rocío, CSIC-Universidad de Sevilla, 41013 Seville, Spain; csalguero-ibis@us.es (C.S.-A.); paulamr1999@gmail.com (P.M.-R.); david.marcilla.sspa@juntadeandalucia.es (D.M.); 2 Centro de Investigación Biomédica en Red de Cáncer, Instituto de Salud Carlos III (CB16/12/00361, CIBERONC-ISCIII), 28029 Madrid, Spain; 3 Department of Normal and Pathological Cytology and Histology, School of Medicine, University of Seville, 41004 Seville, Spain

**Keywords:** solitary fibrous tumor, genetic alterations, gene fusion, molecular diagnostics, risk stratification

## Abstract

**Simple Summary:**

A solitary fibrous tumor (SFT) is a rare mesenchymal neoplasm that can arise at any body location. Local or distant recurrences occur in a significant proportion of cases, but these recurrences are difficult to predict using clinical or pathological features. A specific genetic alteration, the gene fusion *NAB2-STAT6*, is considered to be the defining driver mutation, and different fusion variants seem to account for specific clinical and pathological features, but their prognostic value remains controversial. We inspected a series of 83 SFTs with a high rate of recurrence to evaluate the clinical significance of several potential biomarkers in addition to gene fusion. Our findings confirm previous observations and uncover novel associations of molecular alterations with clinical features, adding additional evidence for their potential application as molecular biomarkers that are helpful to predict the course of the disease.

**Abstract:**

Risk stratification of solitary fibrous tumor (SFT) patients based on clinicopathological features has limited efficacy, especially in predicting late relapse or metastasis. The hallmark alteration of SFT is the gene fusion *NAB2-STAT6*, whose prognostic value remains controversial. As biological knowledge of this entity has increased in recent years, new molecular alterations have emerged that could be helpful to refine current risk models. Here, we evaluated *NAB2-STAT6* fusion variants and other molecular alterations in a series of 83 SFTs that are enriched in progressing cases. Gene fusion variants were identified by targeted RNA-seq in the whole series, whereas *TERT promoter (pTERT)* mutations were inspected by Sanger sequencing in a subset of 18 cases. Immunohistochemical assays were performed to assess BCOR and NTRK expression as well as *P53* mutation status in 45, 44, and 44 cases, respectively. While confirming the associations of gene fusion variants with clinicopathological parameters, our results do not prove their prognostic value. Pan-TRK immunoexpresion correlated with recurrence/progression, P53 staining associated with higher mitotic counts, and *pTERT* mutations were enriched in cases with fatal outcome. An intriguing correlation was found for BCOR protein expression with gene fusion variants, size, and tumor location.

## 1. Introduction

Solitary fibrous tumors (SFT) represent a rare entity of soft tissue tumors of fibroblastic origin with intermediate malignant potential that can arise at any location of the body but are the most common in deep somatic soft tissue sand at body cavity sites (especially the pleura, pelvis, and retroperitoneum) [1,2]. Based on histopathological features, SFTs have been traditionally classified into typical or malignant subtypes, although this distinction does not necessarily correlate with clinical behavior. In consequence, this concept is not referred to in the most recent WHO classification in order to avoid misleading identification of a typical subtype with a benign condition [2]. Currently, risk stratification models incorporating other non-histological parameters (clinical or radiological) are used to predict final patient outcome and adverse events [3]. Among them, the scoring system developed by Demicco and co-workers (m*Demicco*) [4] has been validated in other independent studies and is thus widely used in the clinical setting [5]. This model is based on age, tumor size, mitotic count, and necrosis to predict the risk of metastasis, but it is also accurate when considering different measures of patient outcome such as disease recurrence, which is the parameter used in other risk models [6,7]. Indeed, homogeneous criteria for adverse outcome measure need to be stablished in order to make the performance assessment of different risk systems possible.

Although some of these systems have been proven to have good performance, there is room for improvement, as some cases cannot yet be accurately stratified into high or low risk groups [8]. Recent attempts to refine risk models have explored the possibility of incorporating specific molecular alterations [3]. The molecular hallmark of an SFT is an inversion at chromosomal region 12q13 that generates a gene fusion between NGFI-A-binding protein 2 (*NAB2*) and signal transducer and activator of transcription 6 (*STAT6*), *NAB2-STAT6*, the main driver of the disease. This fusion presents a great variety of fusion variants, but they always produce a chimeric protein in which at least one repressor domain from NAB2 is replaced by a transactivation domain of STAT6, leading to *the* constitutive activation of EGR1-mediated transcription [9,10,11]. Different *NAB2-STAT6* fusion variants are associated with distinct transcriptional profiles, which may result from the presence or absence of specific functional domains in the chimeric protein [12,13]. A few studies have suggested that specific gene fusion variants (retaining only the transactivation domain of STAT6) could account for malignancy and poor patient prognosis [13,14]. However, other authors could not confirm the prognostic value of fusion variants, though there is agreement on their association with other parameters such us patient age, tumor location, or tumor size [15,16,17,18,19,20,21]. Thus, additional molecular features that better perform as prognostic markers are being investigated to improve risk stratification. 

Recent reports describe new promising molecular alterations, namely *P53* and *TERT* promoter mutations, that are associated with adverse clinical evolution [8,15,17,22,23,24,25,26]. However, a broadened catalogue of molecular markers is still warranted to further refine patient stratification. A recent study reported BCOR (Bcl6 interacting co-repressor) overexpression in SFT compared with other types of sarcoma. BCOR protein expression seemed to be associated with malignant SFT, but its informative value regarding patient outcome has not been investigated [27]. Pan-Trk (tropomyosin receptor kinase) protein expression has also been reported in a series of 15 SFT specimens, which could be mainly attributable to NTRK1 overexpression [28]. Among other receptor tyrosine kinases, *NTRK1* is a target gene of EGR1 and displays increased mRNA expression in SFT compared to other tumor types [10]. Downstream kinases induced by NAB2-STAT6 fusion through EGR1 could represent attractive therapeutic targets [10]. Indeed, NTRK inhibitors targeting *NTRK* fusions could also provide clinical benefit in tumors with the overexpression of wild type *NTRK* genes [28].

Herein, we have characterized the different *NAB2-STAT6* fusion variants in a large series of SFTs, which were composed of two independent cohorts, to evaluate the previous reported associations of fusion variants with clinicopathological features. One of the cohorts comprised advanced cases with a long follow-up that progressed after the initial treatment, which should facilitate the identification of specific fusion variants associated with poor prognosis. The second series, with a shorter follow-up, allowed us to increase the sample size in order to analyse associations of gene fusion variants and the clinicopathological parameters. We have also inspected previously proposed molecular alterations, *P53* and p*TERT* mutational status (at positions C228 and C250), and BCOR and NTRK expression to assess their potential prognostic value.

## 2. Materials and Methods

### 2.1. Patients and Sample Series

**Figure 1:**
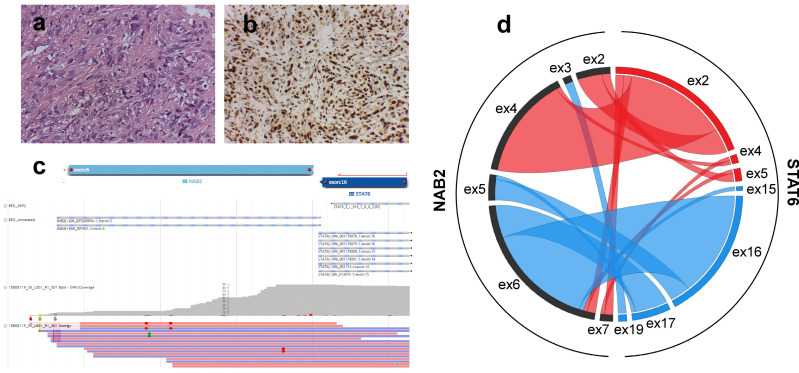
Pathological and molecular characterization of solitary fibrous tumors (SFTs). (**a**) Hematoxylin and eosin staining and (**b**) strong nuclear expression of STAT6 of a meningeal SFT. Magnification 40×. (**c**) Top panel: schematic representation of *NAB2* exon 6 and *STAT6* exon 16 fusion variant and gene specific primer (in red) that covered *STAT6* gene. Middle panel: Sequence alignment between reference and sample sequences. Bottom panel: Mapping of individual reads and coverage supporting the fusion variant between *NAB2* exon6 (NM_005967.3) and *STAT6* exon16 (NM_001178078.1). (**d**) Circos plot showing identified *NAB2-STAT6* gene fusion variants from all the STF cases. Fusion variants classified into STAT6-FULL group are represented by red segments and ribbons, whereas fusion variants classified into STAT6-TAD group are represented in blue. Black segments represent *NAB2* exons.

This study was performed on a patient series composed of two cohorts. The first cohort corresponded to 50 adult patients with advanced and progressing SFTs who were participating in an international single-arm phase II trial by the Spanish Group for Research on Sarcomas (GEIS cohort) [29,30]. All of the patients in this cohort were diagnosed with metastatic or unresectable SFT and had progressed in the previous 6 months of the trial according to either the Choi criteria or the Response Evaluation Criteria in Solid Tumors. Specimens collected from the GEIS cohort corresponded to relapsed tumors. Diagnosis was confirmed by central pathology review, which included the identification of the gene fusion *NAB2-STAT6* by targeted RNA-seq as well as STAT6 immunohistochemistry (IHC) (Figure 1a–c). A second cohort comprised 33 cases (28 primary tumors and 5 relapsed tumors) retrieved from the surgical pathology archives of our institution (Dept. Pathology, Hospital Universitario Virgen del Rocío), who were also subject to molecular confirmation (local cohort). Tissue samples and clinical/follow-up data were obtained from the HUVR-IBiS Biobank (Virgen del Rocio-Institute of Biomedicine of Seville Biobank. Andalusian Public Health System Biobank). All tumors were reviewed by experienced soft tissue pathologists (D. M, E. A.), who evaluated histopathological features that were relevant to the study. Table S1 summarizes all clinicopathological and molecular data for each patient included in the study.

This study was performed following the standard Spanish ethical regulations, and it was approved by the ethics committee of the Hospital Virgen del Rocío de Sevilla and the Fundación Pública Andaluza para la Gestión de la Investigación en Salud de Sevilla (FISEVI), Spain. Written informed consent was obtained from all patients, and all clinical analyses were conducted in accordance with the principles of the Declaration of Helsinki. 

### 2.2. Tissue Microarray (TMA) Construction and Immunohistochemistry (IHC)

IHC for STAT6 was performed on representative formalin-fixed paraffin-embedded (FFPE) tissue sections with anti-STAT6 monoclonal antibody (EP325, Cell Marque, Rocklin, CA, USA). A percentage above 50% of nuclei showing strong STAT6 expression was considered positive [30]. For the remaining IHC markers, representative tumor areas of samples with available material (whole series *n* = 45, GEIS *n* = 31, local *n* = 14) were selected on H&E-stained sections and two 1 mm diameter tissue cores were obtained from each specimen to set up 4 different TMAs. IHC was conducted on TMA sections using the Bond Research Detection System (Leica, Wetzlar, Germany) with a step of heat-induced antigen retrieval (EDTA buffer (pH 9.0)) and using primary antibodies against P53, BCOR, and NTRK. Pan-TRK IHC staining was performed using a pan-TRK antibody, which recognizes TRK proteins including TRK-A, TRK-B, and TRK-C, encoded by the *NTRK1*, *NTRK2*, and *NTRK3* genes, respectively. IHC were performed automatically using the Benchmark ULTRA platform (Roche), following the manufacturer’s instructions. Intensity staining was scored as 0 (negative), 1 (weak), 2 (moderate), or 3 (strong). Extension or percentage of positive cells was scored as 0 (0% of positive cells), 1 (rare to 33%), 2 (34% to 66%), and 3 (67% to 100%). Tissue was given a score, which resulted from multiplying the staining intensity (from 0 to 3) by the extension (from 0 to 3). Thus, sample scores ranged from 0 to 9, as previously described [31]. Samples with nuclear P53 staining and scores ≥2 were classified as positive or high. Low or moderate nuclear BCOR staining was assigned for scores ≤5, whereas samples with scores >5 were classified as high BCOR staining. Samples with pan-TRK IHC score above 0 were classified as positive. The staining patterns of pan-TRK were also recorded as cytoplasmic and/or nuclear [32] Appropriate tissue samples were used as controls for each marker. Images were acquired with a VENTANA iSCAN HT IVD scanner (Roche). Details of the clones, suppliers, dilutions, and cut-off that were used are provided in Table S2. Two expert pathologists (DM and EdA) separately evaluated the IHC sections in a blind manner. 

### 2.3. Targeted RNA-Seq 

Total nucleic acid was extracted from FFPE tissue samples using the Agencourt Formature Kit (A33341; Beckman Coulter, Indianapolis, IN, USA) following the manufacturer’s instructions. The isolated RNA was quantified using the Qubit™ RNA HS Assay kit in combination with a Qubit^®^ 3·0 fluorimeter (Q32852; Thermo Fisher Scientific, Waltham, MA, USA). A total of 200 ng of RNA was used for targeted library preparation, using the Archer™ FusionPlex™ Sarcoma Panel (SK0082; ArcherDX, Boulder, CO, USA) based on a targeted enrichment method called anchored multiplex PCR (AMP). Briefly, RNA was reverse transcribed using random primers, first strand cDNA was synthesized, and RNA quality was assessed using the Archer PreSeq RNA QC assay. After second cDNA strand synthesis, end repair, A-tailing, and adapter ligation, the cDNA was amplified by two rounds of nested PCR using gene-specific primers. Final libraries were quantified with the KAPA Library Quantification Kit (KK4824; KAPA Biosystems, Wilmington, MA, USA) and were pooled to equimolar concentration. Libraries were sequenced on an Illumina MiSeq with MiSeq 300v2 reagents (MS-102-2002; Illumina, San Diego, CA, USA) for paired-end reads, 150 base pair reads, and dual index reads. Samples were multiplexed such that each library was sequenced to at least 1·5 million paired reads or greater in depth. Demultiplexed FASTQ files were analysed using Archer analysis pipeline version 5·1·3. A minimum of five reads with three or more unique start sites spanning the breakpoints were set as cut-off to call fusions.

### 2.4. pTERT Mutation Analysis

Total nucleic acid obtained for the targeted RNA-seq assay was used for this analysis. DNA was quantified using the Qubit™ DNA HS Assay kit in combination with a Qubit^®^ 3·0 fluorimeter (Q32851; Thermo Fisher Scientific, Waltham, MA, USA). The *TERT* promoter amplicon of 163 base pairs (bp) spanning hot-spot mutations at positions 1,295,228 and 1,295,250 on chromosome 5 was amplified using the forward primer 5′-CAGCGCTGCCTGAAACTC-3′ and the reverse primer 5′-GTCCTGCCCCTTCACCTT-3′ using the Amplitaq gold 360 PCR mastermix (Thermo Fisher Scientific, Waltham, MA, USA). Polymerase chain reaction (PCR) was performed with 40–100 ng of DNA in a total volume of 25 μL, with initial denaturation at 95 °C for 7 min followed by 45 cycles with denaturation at 95 °C for 30 s, annealing at 62 °C for 25 s, and extension at 72 °C for 1 min. An amount of 2.5 μL of 360 GC Enhancer was used on each reaction. The amplification product was purified using the QIAquick PCR clean-up kit (Qiagen, Hilden, Germany) according to the manufacturers’ protocols and was subject to bidirectional sequencing using the BigDye terminator v3.1 sequencing kit (Thermo Fisher Scientific, Waltham, MA, USA). Sequencing was performed using an ABI 3500 genetic analyser (Thermo Fisher Scientific, Waltham, MA, USA).

### 2.5. Statistical Analysis

Associations between clinicopathological, immunohistochemical, and molecular variables were assessed by the chi-squared or Fisher’s exact tests for the categorical variables. The Mann–Whitney test was used for analysis of the continuous variables of age and tumor size. Survival curves were estimated using the Kaplan–Meier method, and the differences in survival were evaluated using the long-rank test. All of these statistical analyses were performed using SPSS v25 (SPSS Inc., Chicago, IL, USA) and GraphPad Prism v7 (GraphPad Software, San Diego, CA, USA).

## 3. Results

### 3.1. NAB2-STAT6 Fusion Variants Correlate with Patient Age, Tumor Size and Location

In our study, we included two cohorts of cases (Table 1). A first cohort (GEIS) comprised 50 cases from a phase II clinical trial exploring pazopanib as a first line of therapy in advanced/metastatic SFT [29,30]. All of the patients in this series had progressed before the trial according to either the Choi criteria or the Response Evaluation Criteria in Solid Tumors (RECIST). Therefore, the analyzed tumor specimens in this series corresponded to recurrences occurring before inclusion in the trial. Another cohort of 33 patients (local) with a shorter follow-up, which were diagnosed and treated in our center (not enrolled in the clinical trial), was also included in the study. The median duration of follow-up was 86 and 17 months for the GEIS and local cohorts, respectively. We verified that the local cohort adjusted to the m*Demicco* risk stratification model [4] since the patients at high, intermediate, and low risk behaved as expected. However, the patient stratification of the GEIS cohort did not predict patient outcome accurately when the same model was applied (Figure S1). This might be due to the fact that the m*Demicco* system was validated using primary tumors [4].

All cases were subject to targeted RNA-seq to identify the *NAB2-STAT6* fusion variants (Figure 1). The two most frequent variants were *NAB2ex6-STAT6ex16/17* (*n* = 34, 41%) and *NAB2ex4-STAT6ex2* (*n* = 25, 30.1%), whereas a variety of additional different variants were detected in a lower proportion of cases (*n* = 24, 28.9%) (Figure 1d, Table S3). We reasoned that a cohort of cases with adverse clinical events (GEIS) would enable the identification of relevant associations of gene fusion variants with patient outcome. 

Therefore, we performed both the individual and combined analysis of the cohorts to unveil the associations of the gene fusion variants with clinicopathological parameters (Table 2). Fusion variants were grouped by considering the protein domains of STAT6 that were retained in the predicted chimeric protein, as described by Georgiesh et al. [13]: the STAT6-FULL group comprised those variants with an almost full length *STAT6* sequence, whereas variants that only retained the transactivation domain (TAD) of *STAT6* were included in the STAT6-TAD group. STAT6-TAD and STAT6-FULL variants were equally represented in our whole series (TAD *n* = 43, FULL *n* = 40, 52% and 48%, respectively). Male and females were equally distributed among both groups. The age of the patients at diagnosis in the GEIS cohort harbouring STAT6-FULL variants was significantly higher compared to that of the patients harbouring the STAT6-TAD variants (median age 66 vs. 51 years, Mann–Whitney U test, *p* = 0.001), but this was not the case in the local cohort, though the significance was maintained when testing the aggregated series. Tumors from the GEIS cohort expressing STAT6-FULL variants were larger than those expressing the STAT6-TAD variants, with a median size of 100 mm versus 65 mm (Mann–Whitney U test, *p* = 0.041), although the differences were not statistically significant in the local cohort or the whole series. Regarding tumor location, pleuro-pulmonary SFTs preferentially expressed STAT6-FULL variants in the aggregated analysis (17/21, 81%), while meningeal SFTs mainly expressed STAT6-TAD variants (10/11, 91%), which was more represented in visceral/abdominal and trunk tumors (15/24, 67% and 4/6, 67%, respectively). In contrast, we did not observe preferential expression of any fusion variant in extremity or head and neck tumors. Similar correlations were also observed in the two independent cohorts.

We found no significant association between *NAB2-STAT6* variants and history of recurrence or patient outcome, neither within the cohorts nor in the combined analysis. However, when grouping the *NAB2-STAT6* fusions according to the most frequent variants (namely *NAB2ex6-STAT6ex16/17*, *NAB2ex4-STAT6ex2,* and other), we observed that tumors that eventually relapsed in the whole series preferentially expressed the *NAB2ex6-STAT6ex16/17* variant (*p* = 0.031, Table S3). Moreover, Kaplan–Meier estimates for disease free-survival in the local cohort were shorter, though not significant, for the cases with STAT6-TAD variants compared to those with STAT6-FULL variants (mean, 49.8 versus 67.8, long-rank test, *p* = 0.331, Figure S2). 

### 3.2. Analysis of Additional Molecular Markers 

**Figure 2:**
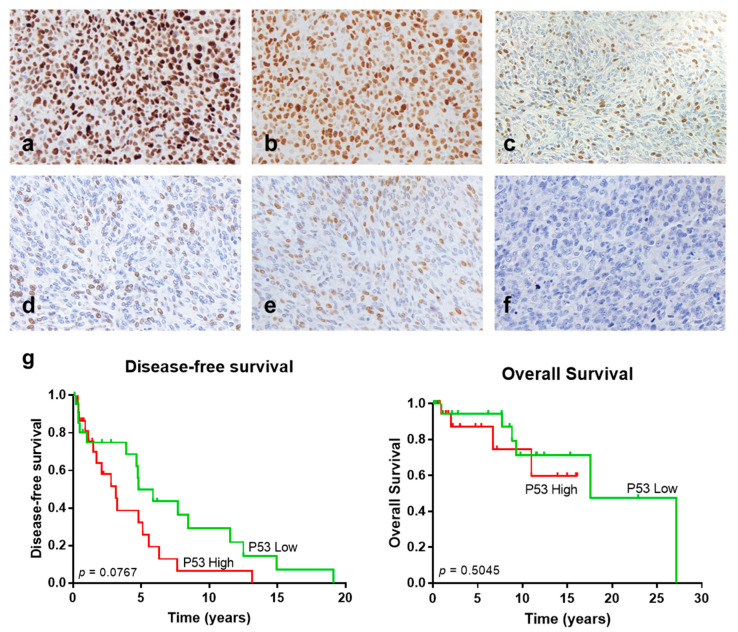
Immunohistochemistry of P53, BCOR, and pan-TRK in solitary fibrous tumors (SFTs). Representative images (40×) of high (**a**) and low (**d**) nuclear P53 staining; high (**b**) and low (**e**) nuclear BCOR staining and positive (nuclear) (**c**) and negative (**f**) pan-TRK staining. (**g**) Kaplan–Meier survival curves of disease-free survival (**left**) and overall survival (**right**) stratified based on P53 IHC staining in SFTs.

Several studies have shown that the *P53* mutation could contribute to malignant SFT transformation [15,33,34]. Thus, we studied P53 expression by IHC as a surrogate marker for *P53* mutational status in a subset of cases with available material in our series (30 cases from the GEIS cohort and 14 cases from the local series) (Table 3). We found that P53 staining correlated with higher mitotic count, but no association was observed with other clinicopathological parameters nor fusion variants. A total of 16 out of 24 (67%) of the SFTs with a high mitotic index (≥4 mitotic count per 10 high power fields, HPFs) exhibited high P53 staining (Figure 2a), while 13 of 18 (72%) SFTs with a low mitotic index (<4 mitotic count per 10 HPFs) showed low or absent expression (Figure 2d) (Fisher’s exact test, *p* = 0.0278). We did not find any correlation between P53 staining and adverse clinical events (Table 3), although cases with high P53 staining showed a shorter (not significant) disease-free survival (DFS) time (long-rank test, *p* = 0.0767) (Figure 2g). Nevertheless, these results should be interpreted with caution since most of the specimens corresponded to recurrences.

We also investigated BCOR IHC expression (Table 3), which has recently been reported to be overexpressed in renal malignant SFTs showing an undifferentiated/small round cell phenotype [27]. High BCOR IHC expression (Figure 2b) correlated with tumor size and location. The tumor size of SFTs with low BCOR staining (Figure 2e) ranged from 30 to 270 mm (median = 110 mm, mean = 125.7 mm), whereas tumors with a high BCOR staining ranged from 15 to 130 mm (median = 60 mm, mean = 62 mm, Mann–Whitney test, *p* = 0.0013). Preferential high BCOR expression was observed in head/neck, trunk, meningeal, and extremity SFTs (80%, 67%, 623% and 60%, respectively). In contrast, BCOR was usually poorly expressed in abdominal/visceral and pleuro-pulmonary SFTs (Table 3, X^2^ test, *p* = 0.0221). Interestingly, most of the cases with high BCOR IHC staining corresponded to those expressing the variant STAT6-TAD (15/18, 83.3%, Fisher’s exact test, *p* = 0.0027) (Table 3). No association was found between BCOR staining and clinical behaviour. 

Next, pan-TRK immunostaining was analysed in the same subset of cases. Robinson et al. [10] showed that NTRK1, a target gene of EGR1, displays increased mRNA expression in SFTs compared to other tumor types. We observed moderate to low nuclear and/or cytoplasmic expression or absent expression of NTRK proteins in our series (Figure 2c,f and Figure S3). Remarkably, most of the cases showed nuclear pan-NTRK staining (25/31 cases, 80.6%); while cytoplasmic and/or cytoplasmic and nuclear staining was observed in 6/31 cases (19.4%) (Table S1). No evident correlation with the pathological parameters was identified (Table 3). However, pan-TRK expression was associated with history of recurrence or progression (Fisher’s exact test, *p* = 0.0078), and all cases with fatal outcome displayed pan-TRK positivity (10/10, 100%). No preferential pan-TRK expression was observed in cases with specific fusion variants.

**Figure 3:**
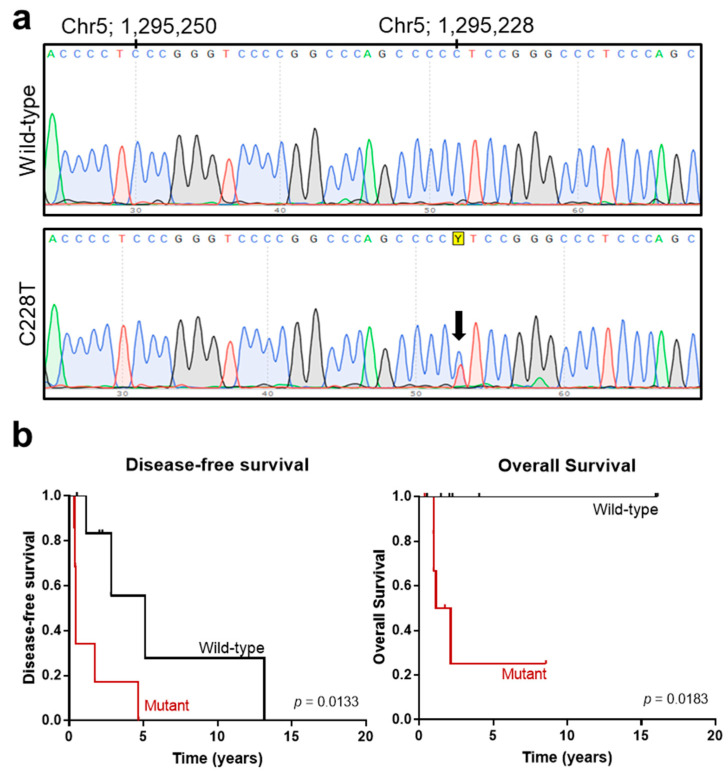
*TERT* gene promoter mutational analysis. (**a**) Representative chromatograms of wild-type (**top**) and C228T heterozygous mutation (**lower**) found in solitary fibrous tumors (SFTs). Yellow Y represents the standard abbreviations to represent C or T. (**b**) Kaplan–Meier survival curves of disease-free survival (**left**) and overall survival (**right**) stratified based on *TERT* promoter mutational status in SFTs.

Finally, we evaluated *pTERT* mutational status, which has been proposed as a marker of malignant features and poor clinical outcome in several studies [4,8,15,17,22,35]. Only 18 samples rendered DNA that could be PCR-amplified. A total of 10 cases corresponded to the GEIS cohort, and 8 cases corresponded to the local SFT cohort. We studied *pTERT* hot-spot mutations at positions 1,295,228 (C228) and 1,295,250 (C250). A total of 9 out of 18 cases were mutated at position C228 (Figure 3), and only one case presented the alteration in both alleles (case #38, Table S1). No mutations were detected in position C250. The median age at diagnosis for non-mutated *pTERT* cases was 49 years of age, while the median for the *pTERT*-mutated cases was 63 years of age. Regarding tumor location, the four pleuro-pulmonary cases presented *pTERT* mutations, whereas the abdominal/visceral specimens predominantly showed a non-mutated *pTERT* status. Moreover, three out of four cases with necrosis presented a *pTERT* mutation status. We did not find a correlation between *pTERT* mutation status and gender, tumor, size or mitotic counts. However, most of the cases with STAT6-FULL fusion variants presented *pTERT* mutations (8/10, 80%) in contrast to STAT6-TAD variants, which correlated with wild-type *pTERT* (7/9, 88%) (Table 4; Fisher’s exact test, *p* = 0.0152). Remarkably, all of the patients who died of the disease presented *pTERT* mutations (4/4, 100%), while only 3 out of 10 (30%) patients alive at final follow-up time harboured the mutation (Fisher’s exact test, *p* = 0.0699). Moreover, we observed a decrease in both disease/progression-free survival and overall survival time for patients with *pTERT* mutations (long-rank test, *p* = 0.0133 and *p* = 0.0183, respectively) (Figure 3b). As noted before for BCOR, survival analyses should be interpreted carefully because 10 out 18 samples corresponded to relapsed tumors.

## 4. Discussion

Our results confirm previous studies reporting the association of *NAB2-STAT6* fusion variants with clinicopathological features of SFTs [15,16,17,18,19,20,21]. We found that STAT6-FULL variants were preferentially expressed in older patients, slightly larger tumors, and pleuro-pulmonary location. Conversely, the expression of STAT6-TAD variants correlated with younger patients, smaller tumors, and predominant meningeal location. However, we did not find associations of the gene fusion variants with mitotic count or necrosis, traditional parameters that are used to classify SFTs into malignant or typical. Both the long follow-up and severity of the disease in the GEIS cohort enabled the recording of fatal outcomes, thus allowing us to test whether exitus was related to gene fusion variants. In spite of using a series with advanced cases and long follow-up (GEIS cohort), we found no correlation of gene fusion variants with the final patient outcome nor did the STAT6-FULL/TAD variants predict adverse clinical events when analysing a whole series enriched in advanced cases. A trend towards poor prognosis could be observed for the *NAB2ex6-STAT6ex16/17* variants (Table S3), which is in agreement with the studies that report a prognostic impact of fusion variants on clinical outcome [13,14]. Taken together, our observations point towards a marginal role as to if any of the fusion variants determine the clinical behaviour of the tumor. Nevertheless, a recent report suggested that biological properties are somehow determined by fusion variants, i.e., through the different modulation of EGR1-dependent gene expression [12]. Controversy about the prognostic value of *NAB2-STAT6* fusion variants brings about the necessity of a deep molecular characterization of SFTs to identify potential biomarkers that better predict patient outcome. In recent years, *pTERT* and *P53* mutation status have emerged as potential prognostic biomarkers. Though we inspected a low number of cases for *pTERT* mutations, our results support its potential impact on clinical parameters and prognosis. Interestingly, the only sample with homozygous *pTERT* mutation corresponded to an SFT of the extremity, which is in accordance with a report describing the higher frequency of the *pTERT* homozygous mutation at this specific location [36]. The association of the *pTERT* mutation with STAT6-FULL variants (which correspond to *NAB2ex4-STAT6ex2* fusion variants in our tested cases) has not been described before and deserves confirmation in a larger series of cases, but it is compatible with its higher occurrence in older patients. 

Regarding P53 expression, our results partially agree with previous reports. We did observe correlation of P53 IHC expression with mitotic count, a parameter denoting malignancy, as described by Park et al. [15], but the lack of association with other pathologic parameters does not parallel the results in the study by Schirosi et al. [26], though this study is focused on pleuro-pulmonary SFT. Shorter disease-free survival for cases with P53 overexpression was described in the same report and in other study [17,26], and our analysis revealed a similar trend, but it was one that was not statistically significant. Since the cohort from the GEIS trial consisted of relapsed cases, we expected a high incidence of the secondary alterations with potential prognostic significance. However, the proportion of cases with high P53 staining was the same in both the GEIS and local cohorts (50% of cases). Regarding p*TERT* status, the GEIS cohort showed a higher proportion of mutated cases (6/10, 60%) compared to the local series (3/8, 37.5%), but this difference did not reach statistical significance. Akaike et al. [17] proposed a model for the clonal evolution in the differentiation process of SFTs that involves *pTERT* and *P53* mutations that occur at different stages. Our series included four cases diagnosed as dedifferentiated SFTs, with two of them having available data for P53, *pTERT* mutation and clinical follow-up (cases #20 and #38). Case #20 showed no *pTERT* mutation and displayed high P53 staining, with no disease progression occurring until the end of follow-up (26 months). Case #38, which showed the *pTERT* mutation accompanied by high P53 expression, suffered a relapse after 5 months from diagnosis and died of the disease 6 months later. Interestingly, case #38 was classified into the low-risk category attending to the m*Demicco* score, thus highlighting the utility of these molecular markers for the accurate assessment of tumor behavior in particular cases.

Our study revealed that BCOR immunoreactivity is frequent in SFTs and that it is tightly associated with fusion variant and tumor size and location. Argani et al. [27] recently reported intense BCOR protein expression in five index cases of renal malignant SFTs. They also showed that BCOR nuclear labelling was even stronger than STAT6 staining in these index cases and in a subset of 39 cases of extrarenal SFTs. A recent report also describes that BCOR is more upregulated in *NAB2ex6-STAT6ex16/17* cases than it is in *NAB2ex4-STAT6ex2* cases, which was determined by RNA-seq [12]. The former study also revealed that BCOR was overexpressed in SFTs compared to other types of sarcoma. Moreover, BCOR protein expression was associated with malignant SFTs when defined as attending to pathologic criteria [27]. However, they did not observe preferential BCOR staining for specific tumor locations. Further studies in independent series are warranted to confirm our observations, or alternatively, analyses of transcriptomic data for tumors with different location or fusion variants, since BCOR overexpression seems to occur at the mRNA level [27,37]. It is tempting to speculate that *NAB2ex6-STAT6ex16/17* variants (STAT6-TAD), usually non-pleuropulmonary, may induce a transcriptomic profile in which BCOR could be a specific direct or indirect target. Even though BCOR expression did not demonstrate any prognostic value, it may be useful as an additional IHC marker for SFT, providing additional information given its strong association with other parameters.

We found a striking association between pan-TRK positivity and poor patient outcome, which was not accompanied by other relevant correlations. A recent study by Kao et al. [28] investigating NTRK upregulation in tumors with BCOR and YWHAE rearrangements reported pan-TRK expression in a control group of SFT cases (15/15, 100%), with diverse risk of malignancy and different anatomic locations. A total of 10 out of 15 cases also showed BCOR IHC expression. In our study, we observed 31/44 SFT cases that were positive for pan-TRK staining (70.5%). This discrepancy in the proportion of SFTs positive for pan-TRK may be due to the use of TMA in our study instead of whole tissue sections. The same inconsistency was indeed reported in the mentioned study for the IHC analysis of pan-TRK in synovial sarcomas. They also determined that pan-TRK protein overexpression in SFT was mainly attributable to the upregulation of *NTRK1* mRNA. Since *NTRK1* is a target of *EGR1*, the central hub involved in NAB2-STAT6-dependent deregulation of gene expression [10,38], our results may suggest that an increased EGR1 transcriptional program could lead to a more aggressive tumor phenotype. This correlation has been not reported before and deserves further confirmation. In vitro functional assays with cell line models could help to assess this possibility.

## 5. Conclusions

In conclusion, our study did not confirm a clear prognostic value for *NAB2-STAT6* fusion variants in SFTs, while *P53* and *pTERT* mutations could be valuable ancillary markers in the clinical setting. Our investigation on new molecular features awaits further confirmation, but it may offer new markers that could be useful as surrogates for tumor characterization (BCOR expression) and relevant for patient risk stratification (NTRK expression). 

## Figures and Tables

**Table 1:** Clinicopathological features and outcome of patients with solitary fibrous tumors in each cohort.

Variables	GEIS Cohort	Local Cohort	Whole Series
**Sex**	*n* = 50	*n* = 31	*n* = 81
Male	22 (44)	13 (42)	35 (43)
Female	28 (56)	18 (58)	46 (57)
**Age (years)**	*n* = 50	*n* = 30 ^a^	*n* = 80 ^a^
Mean ± SD	54.5 ± 16.5	55.8 ± 13.5	55.1 ± 15.4
Median (Range)	54 (20–87)	56 (27–79)	55 (20–87)
**Size, mm**	*n* = 34 ^a^	*n* = 21 ^a^	*n* = 55 ^a^
Mean ± SD	95.2 ± 59.7	90.9 ± 51.0	93.5 ± 56.1
Median (Range)	85 (15–270)	95 (15–210)	85 (15–270)
**Location**	*n* = 50	*n* = 31 *	*n* = 81
Abdominal/Visceral	11 (22)	13 (42)	24 (30)
Pleuro-pulmonary	13 (26)	8 (26)	21 (26)
Meningeal	9 (18)	2 (6)	11 (14)
Extremity	6 (12)	4 (13)	10 (12)
Head and Neck	7 (14)	2 (6)	9 (11)
Trunk	4 (8)	2 (6)	6 (7)
**Mitotic count (/10 HPFs)**	*n* = 50	*n* = 29 ^a^	*n* = 79 ^a^
Mean ± SD	5.5 ± 8.3	3.8 ± 5.0	4.9 ± 7.3
Median (Range)	3 (0–48)	2 (0–17)	3 (0–48)
<4	27 (54)	20 (69)	47 (59)
≥4	23 (46)	9 (31)	32 (41)
**Necrosis**	*n* = 49 ^a^	*n* = 25 ^a^	*n* = 74 ^a^
Absent	41 (84)	21 (84)	62 (84)
Present	8 (16)	4 (16)	12 (16)
**Follow-up for OS**			
Median, months (Range)	86 (9–326)	17 (1–132)	48 (1–326)
**Follow-up for DFS**			
Median, months (Range)	47 (4–230)	17 (1–74)	28 (1–229)
**Recurrence/progression**	*n* = 49 ^a^	*n* = 27 ^a^	*n* = 76 ^a^
No	0 (0)	21 (78)	21 (28)
Yes	49 (100)	6 (22)	55 (72)
**Outcome**	*n* = 49 ^a^	*n* = 27 ^a^	*n* = 76 ^a^
Alive	33 (67)	26 (96)	59 (78)
Dead of disease	16 (33)	1 (4)	17 (22)

Data are given as number (%) unless otherwise indicated. ^a^ Frequencies not summing to column total indicate missing data. (*) The sum of percentages is 99 because of the value rounding.

**Table 2:** Associations of *NAB2-STAT6* gene fusion variants with clinicopathological parameters.

	GEIS Cohort			Local Cohort			Whole Series		
*NAB2-STAT6* Variant	TAD	FULL	*p* Value	TAD	FULL	*p* Value	TAD	FULL	*p* Value
**Relative frequency**									
N	25 (50)	25 (50)		18 (55)	15 (45)		43 (52)	40 (48)	
**Sex**			0.612			0.722			0.8241
Male	11 (50)	11 (50)		6 (46)	7 (54)		17 (49)	18 (51)	
Female	14 (50)	14 (50)		10 (56)	8 (44)		24 (52)	22 (48)	
**Age (years)**			**0.001**			0.448			**0.001**
Mean (± SD)	46.7 ± 15.0	62.3 ± 14.8		53.9 ± 13.4	57.6 ± 13.9		49.6 ± 14.7	61.0 ± 14.1	
Median (Range)	51 (20–79)	66 (31–87)		54 (27–79)	58 (32–77)		52 (20–79)	65 (31–87)	
**Size (mm)**			**0.041**			0.468			0.225
Means ± SD	76.0 ± 51.9	114.4 ± 63.6		98.3 ± 55.9	84.1 ± 47.8		84.6 ± 53.5	101.5 ± 58.1	
Median (Range)	65 (15–200)	100 (30–270)		98 (15–210)	75 (35–200)		73 (15–210)	95 (30–270)	
**Location**			**0.048**			**0.019**			**0.0029**
Abdominal/Visceral	4 (36)	7 (64)		11 (85)	2 (15)		15 (67)	9 (37)	
Pleuro-pulmonary	3 (23)	10 (77)		1 (12)	7 (88)		4 (19)	17 (81)	
Meningeal	8 (89)	1 (11)		2 (100)	0 (0)		10 (91)	1 (9)	
Extremity	3 (50)	3 (50)		1 (25)	3 (75)		4 (40)	6 (60)	
Head and Neck	4 (57)	3 (43)		1 (50)	1 (50)		5 (56)	4 (44)	
Trunk	3 (75)	1 (25)		1 (50)	1 (50)		4 (67)	2 (33)	
**Necrosis**			0.327			0.645			0.2159
Absent	22 (54)	19 (46)		10 (55)	8 (45)		34 (55)	28 (45)	
Present	3 (51)	5 (49)		7 (40)	4 (60)		4 (33)	8 (67)	
**Mitotic count/10 HPFs**			0.571			0.717			0.6543
<4	15 (56)	12 (44)		10 (56)	8 (44)		26 (55)	21 (45)	
≥4	10 (43)	13 (57)		7 (64)	4 (36)		16 (50)	16 (50)	
**Recurrence/progression**			-			0.165			0.445
No	0 (0)	0 (0)		9 (43)	12 (57)		9 (43)	12 (57)	
Yes	25 (51)	24 (49)		5 (83)	1 (17)		30 (55)	25 (44)	
**Outcome**			1			1			1
Alive	17 (52)	16 (48)		13 (50)	13 (50)		30 (51)	29 (49)	
Dead of disease	8 (50)	8 (50)		1 (100)	0 (0)		9 (53)	8 (47)	

Data are given as number (%) unless otherwise indicated. Significant *p* values are indicated in bold.

**Table 3:** Associations of IHC markers with clinicopathological parameters and gene fusion variants.

	P53 IHC (*n* = 44)			BCOR IHC (*n* = 45)			pan–TRK IHC (*n* = 44)		
	Low	High	*p* Value	Low	High	*p* Value	Negative	Positive	*p* Value
	*n* = 22	*n* = 22		*n* = 27	*n* = 18		*n* = 13	*n* = 31	
**Gender**			0.7546			0.5372			0.4964
Male	7 (44)	9 (56)		9 (53)	8 (47)		6 (37)	10 (66)	
Female	15 (54)	13 (46)		18 (64)	10 (36)		7 (25)	21 (75)	
**Age, years**			0.3126			0.3248			0.0679
Mean ± SD	48.7 ± 14.9	53.9 ± 17.2		53.2 ± 16.9	48.7 ± 14.5		44.7 ± 15.3	54.1 ± 15.9	
Median (Range)	50 (28–87)	50.5 (20–79)		52 (20–87)	45 (27–79)		40 (20–78)	52 (27–87)	
**Size (mm)**			0.2988			**0.0013**			0.7031
Mean ± SD	108.9 ± 57.9	91.9 ± 67.5		125.7 ± 62.8	62.0 ± 33.1		89.6 ± 49.0	106.9 ± 68.4	
Median (Range)	100 (27–210)	85 (15–270)		110 (30–270)	60 (15–130)		98 (15–200)	90 (27–270)	
**Locations**			0.1915			**0.0221**			0.1894
Abdominal/Visceral	6 (55)	5 (45)		8 (73)	3 (27)		2 (18)	9 (82)	
Pleuro–pulmonary	8 (62)	5 (38)		12 (92)	1 (8)		6 (46)	7 (54)	
Meningeal	3 (37)	5 (63)		3 (37)	5 (63)		1 (12)	7 (88)	
Extremity	0 (0)	4 (100)		2 (40)	3 (60)		0 (0)	4 (100)	
Head and Neck	4 (80)	1 (20)		1 (20)	4 (80)		3 (60)	2 (40)	
Trunk	1 (33)	2 (67)		1 (33)	2 (67)		1 (33)	2 (67)	
**Mitotic counts/10 HPFs**			**0.0278**			0.1678			0.4832
<4	13 (72)	5 (28)		17 (57)	13 (43)		6 (33)	12 (67)	
≥4	8 (33)	16 (67)		9 (82)	2 (18)		5 (21)	19 (79)	
**Necrosis**			0.4882			0.1678			0.2268
Absent	14 (48)	15 (52)		17 (57)	13 (43)		5 (17)	24 (83)	
Present	7 (64)	4 (36)		9 (82)	2 (18)		4 (36)	7 (64)	
**Fusion Variant**			0.364			**0.0027**			0.522
STAT6–TAD	14 (58)	10 (42)		10 (40)	15 (60)		6 (25)	18 (75)	
STAT6–FULL	8 (40)	12 (60)		17 (85)	3 (15)		7 (35)	13 (65)	
**Recurrence/progression**			>0.9999			0.7323			**0.0078**
No	6 (55)	5 (45)		6 (55)	5 (45)		7 (64)	4 (36)	
Yes	16 (48)	17 (52)		21 (62)	13 (38)		6 (18)	27 (82)	
**Outcome**			0.7205			0.7323			**0.0212**
Alive	16 (47)	18 (53)		21 (62)	13 (38)		13 (38)	21 (62)	
Died of disease	6 (60)	4 (40)		6 (55)	5 (45)		0 (0)	10 (100)	

Data are given as number (%) unless otherwise indicated. Significant *p* values are indicated in bold.

**Table 4:** Associations of *TERT* gene promoter mutations with clinicopathological parameters and gene fusion variants.

	*pTERT* Mutational Status		
	Wild-Type	Mutant	*p* Value
	*n* = 9	*n* = 9	
**Gender**			0.6372
Male (%)	6 (60)	4 (40)	
Female (%)	3 (37)	5 (63)	
**Age, years**			0.0876
Mean ± SD	48.7 ± 17.1	63.1 ± 16.5	
Median (Range)	49 (20–70)	64.5 (31–87)	
**Size (mm)**			1
Mean ± SD	122.0 ± 94.4	104.4 ± 46.8	
Median (Range)	120 (15–270)	100 (37–165)	
**Locations**			0.0537
Abdominal/Visceral (%)	6 (86)	1 (14)	
Pleuro-pulmonary (%)	0 (0)	4 (100)	
Meningeal (%)	2 (67)	1 (33)	
Extremity (%)	0 (0)	1 (100)	
Head and Neck (%)	0 (0)	1 (100)	
Trunk (%)	1 (100)	0 (0)	
**Mitotic counts/10 HPFs**			0.6199
<4 (%)	2 (33)	4 (67)	
≥4 (%)	7 (58)	5 (42)	
**Necrosis**			0.5846
Absent (%)	6 (50)	6 (50)	
Present (%)	1 (25)	3 (75)	
**Fusion variant**			**0.0152**
STAT6-TAD	7 (88)	1 (12)	
STAT6-FULL	2 (20)	8 (80)	
**Recurrence/progression**			0.5594
No (%)	3 (75)	1 (25)	
Yes (%)	4 (40)	6 (60)	
**Outcome**			0.0699
Alive (%)	7 (70)	3 (30)	
Died of disease (%)	0 (0)	4 (100)	

Data are given as number (%) unless otherwise indicated. Significant *p* values are indicated in bold.

## Data Availability

The data presented in this study are available upon request from the corresponding author.

## Supplementary Materials

The following are available online at https://www.mdpi.com/article/10.3390/cancers13205237/s1, Figure S1: Patient risk stratification in GEIS and local cohorts attending to the m*Demicco* system; Figure S2: Kaplan–Meyer estimates for fusion variants in the local cohort; Figure S3: Representative immunohistochemical stains for pan-TRK; Table S1: Summary of clinicopathological and molecular data from all 83 solitary fibrous tumor (SFTs) cases; Table S2: Antibodies, suppliers, dilutions, and scoring; Table S3: Associations of *NAB2-STAT6* fusion variants with clinicopathological parameters.
