# Supplementary material for: Evaluation of NAB2-STAT6 Fusion Variants and Other Molecular Alterations as Prognostic Biomarkers in a Case Series of 83 Solitary Fibrous Tumors
Source: Cancers (Basel). 2021 Oct 19;13(20):5237. doi: 10.3390/cancers13205237 (PMC8534228; doi:10.3390/cancers13205237)
Supplement: Supplementary file 1 [file cancers-13-05237-s001.zip › Figure S1.pdf]

	Low	Intermediate	High	p value
**GEIS cohort, n=37**	n=20	n=8	n=9	
**Recurrence/progression**				
No (%)	0 (0)	0 (0)	0 (0)	-
Yes (%)	20 (54.1)	8 (21.6)	9 (24.3)	
**Outcome**				
Alive (%)	15 (60)	4 (16)	6 (24)	0.4417
Died of disease (%)	5 (41.7)	4 (33.3)	3 (25)	
**Local cohort, n=18**	n=11	n=5	n=2	
**Recurrence/progression**				**0.0022**
No (%)	11 (73.3)	4 (26.7)	0 (0)	
Yes (%)	0 (0)	1 (33.3)	2 (66.7)	
**Outcome**				**0.0145**
Alive (%)	11 (64.7)	5 (29.4)	1 (5.9)	
Died of disease (%)	0 (0)	0 (0)	1 (100)	

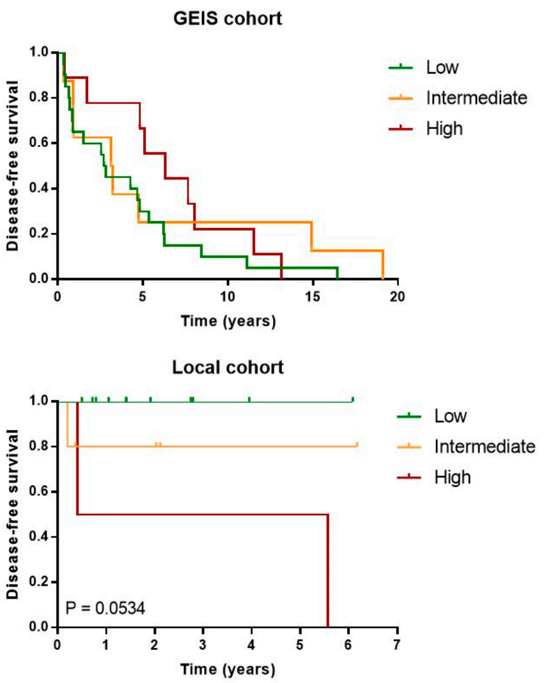

Figure S1. Patient risk stratification in GEIS and local cohorts attending to the m*Demicco* system. Patients with age, tumor size, mitotic count and necrosis available data were classified into three risk groups (low, intermediate and high) according to m*Demicco* risk stratification model. Kaplan-Meier survival curves of disease-free survival of GEIS (top) and local (bottom) cohorts, stratified based on risk groups.
