# Supplementary material for: Evaluation of NAB2-STAT6 Fusion Variants and Other Molecular Alterations as Prognostic Biomarkers in a Case Series of 83 Solitary Fibrous Tumors
Source: Cancers (Basel). 2021 Oct 19;13(20):5237. doi: 10.3390/cancers13205237 (PMC8534228; doi:10.3390/cancers13205237)
Supplement: Supplementary file 1 [file cancers-13-05237-s001.zip › Figure S2.pdf]

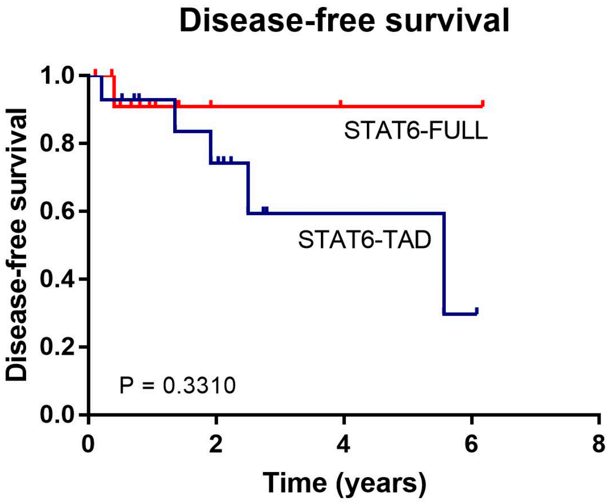

Figure S2. Kaplan-Meyer estimates for fusion variants in the local cohort. Kaplan-Meier survival curves of disease-free survival of local cohorts, stratified based on fusion variants.
