# Supplementary material for: Evaluation of NAB2-STAT6 Fusion Variants and Other Molecular Alterations as Prognostic Biomarkers in a Case Series of 83 Solitary Fibrous Tumors
Source: Cancers (Basel). 2021 Oct 19;13(20):5237. doi: 10.3390/cancers13205237 (PMC8534228; doi:10.3390/cancers13205237)
Supplement: Supplementary file 1 [file cancers-13-05237-s001.zip › Figure S3.pdf]

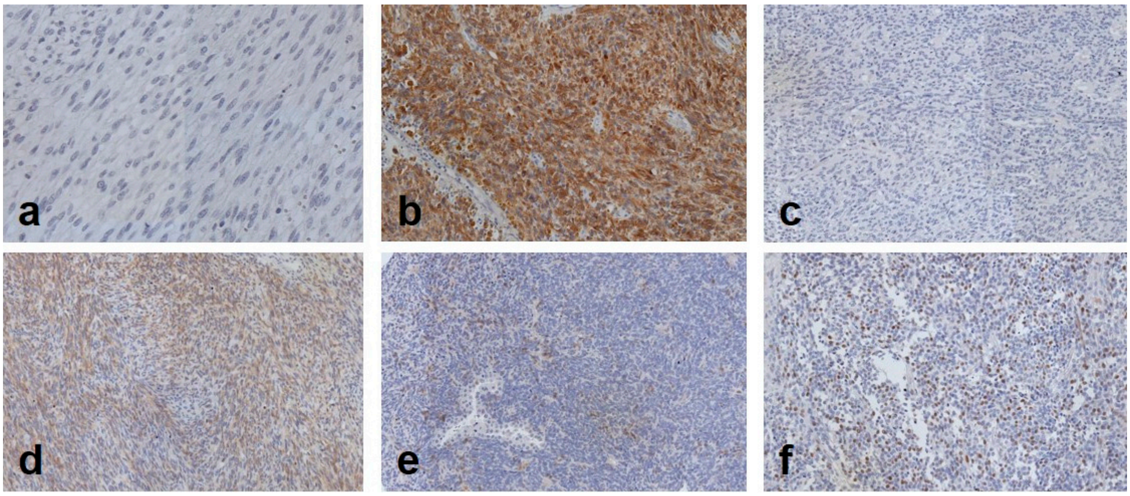

Figure S3. Representative immunohistochemical staining for pan-NTRK. (**a**) Negative control: endometrial stromal sarcoma with *ZC3H7B-BCOR* gene rearrangement, Magnitude 40X; (**b**) Positive control: fusocelular mesenchymal neoplasia with *NTRK1-TPM3* gene fusion, Magnitude 20X; (**c**) Negative case: pleuro-pulmonary Solitary Fibrous Tumor (SFT) (#57); (**d**) Cytoplasmic staining: abdominal dedifferentiated SFT (#80), (**e**) Nuclear and cytoplasmic staining: extremity SFT (#51); (**f**) Nuclear staining: meningeal SFT (#50), Magnitude 20X. SFT case numbers correspond to those specified in Table S1.
