# Supplementary material for: Evaluation of NAB2-STAT6 Fusion Variants and Other Molecular Alterations as Prognostic Biomarkers in a Case Series of 83 Solitary Fibrous Tumors
Source: Cancers (Basel). 2021 Oct 19;13(20):5237. doi: 10.3390/cancers13205237 (PMC8534228; doi:10.3390/cancers13205237)
Supplement: Supplementary file 1 [file cancers-13-05237-s001.zip › Table S1.pdf]

Table S1. Summary of clinicopathological and molecular data from all 83 solitary fibrous tumor (SFT) cases

#	Cohort	Specimen type	Age (years)	Gender	Location	Size (mm)	Mitotic counts	Necrosis	STAT6 IHC	P53 IHC	BCOR IHC	Pan-TRK IHC	*pTERT* status	*NAB2-STAT6* fusion variant	*NAB2-STAT6* group	Relapse or progression	DFS	Exitus	OS	m*Demicco* risk / CNS-G grade
1	L	P	38	F	PP	70	NA	NA	+	1.5	6	0	NA	7-1int	FULL	No	1.3	No	1.3	NA
2	L	P	45	F	PP	140	1	yes	+	0	2	1.5 (N)	NA	6-17	TAD	Yes	2.5	No	2.5	Int
3	L	P	65	F	PP	100	0	yes	NA	0	1	0	C228T	4-2	FULL	No	4.4	No	4.4	Int
4	L	P	52	M	PP	200	15	yes	+	9	4	0	NA	4-2	FULL	Yes	4.9	No	13.3	High
5	L	P	56	F	Ext	45.5	0	No	+	NA	NA	NA	NA	2-2	FULL	No	6.0	No	6.0	Low
6	L	P	52	M	Vis	15	1	NA	+	3.5	6	0	wt	6-17	TAD	No	6.3	No	6.3	NA
7	L	P	32	F	PP	75	NA	NA	+	3	0	0	NA	4-2	FULL	No	8.0	No	8.0	NA
8	L	P	40	M	H&N	100	0	No	NA	1	9	0	NA	5-16	TAD	No	8.6	No	8.6	Low
9	L	P	41	F	Abd	70	0	No	+	4	6	2 (C)	NA	5-17	TAD	No	9.5	No	9.5	Low
10	L	P	60	M	Trunk	NA	0	No	+	NA	NA	NA	NA	7-1int	FULL	No	9.7	No	9.7	NA
11	L	P	77	F	Ext	NA	NA	NA	NA	NA	NA	NA	NA	4-2	FULL	No	11.5	No	11.5	NA
12	L	P	55	M	H&N	35	1	No	+	NA	NA	NA	NA	4-4	FULL	No	12.6	No	12.6	Low
13	L	R	60	M	Vis	NA	6	NA	+	NA	NA	NA	NA	6-17	TAD	Yes	16.3	No	28.4	NA
14	L	P	43	M	Vis	95	0	No	+	NA	NA	NA	NA	4-2	FULL	No	16.9	No	16.9	Low
15	L	P	49	M	PP	98	3	No	+	5	3.5	0	NA	4-2	FULL	No	17.0	No	17.0	Low
16	L	P*	62	F	Abd	NA	NA	NA	+	NA	NA	NA	NA	3-19	TAD	Yes	23.0	No	38.0	NA
17	L	P	68	M	PP	37	1	No	+	NA	NA	NA	NA	4-2	FULL	No	23.0	No	23.0	Low
18	L	P	27	M	Vis	120	7	No	NA	NA	NA	NA	wt	6-17	TAD	No	24.4	No	24.4	Int
19	L	P	56	F	Abd	210	3	Yes	+	1	5	2.5 (N)	NA	5-16	TAD	No	25.4	No	25.4	High
20	L	P*	49	F	Vis	130	17	NA	+	5	6	0	wt	6-16	TAD	No	26.8	No	26.8	NA
21	L	R	68	F	Abd	NA	2	NA	+	NA	NA	NA	NA	6-17	TAD	Yes	30.0	No	90.9	NA
22	L	P	62	F	Ext	95	0	No	+	NA	NA	NA	NA	5-16	TAD	No	32.9	No	32.9	Low
23	L	P	48	F	Vis	63	4	No	+	0.5	1.5	2.5 (N)	NA	6-16	TAD	No	33.5	No	33.5	Low
24	L	P	71	F	Ext	50	0	No	+	NA	NA	NA	NA	2-5	FULL	No	47.4	No	47.4	Low
25	L	R	42	F	Men	NA	9	No	NA	3	9	1.5 (N)	NA	6-16	TAD	Yes	66.8	Yes	131.9	CNS-G III
26	L	P	79	F	Trunk	40	1	No	NA	NA	NA	NA	NA	3-19	TAD	No	73.0	No	73.0	Low
27	L	P	69	F	PP	120	3	No	NA	1	1	0.5 (N)	NA	4-2	FULL	No	74.1	No	74.1	Int
28	L	R	NA	NA	NA	NA	0	No	+	NA	NA	NA	NA	6-16	TAD	NA	NA	NA	NA	NA
29	L	R	NA	F	NA	NA	12	No	NA	NA	NA	NA	C228A	4-2	FULL	NA	NA	NA	NA	NA
30	L	P*	74	M	Vis	NA	14	yes	+	NA	NA	NA	C228T	4-2	FULL	NA	NA	NA	NA	NA
31	L	P	63	M	Abd	NA	5	No	+	NA	NA	NA	wt	6-16	TAD	NA	NA	NA	NA	NA

32	L	P	NA	NA	Abd	NA	3	No	+	NA	NA	NA	NA	6-15	TAD	NA	NA	NA	NA	NA
33	L	P	70	M	Men	NA	3	No	+	NA	NA	NA	wt	6-16	TAD	NA	NA	NA	NA	CNS-G III
34	G	R	64	M	PP	165	0	No	+	NA	NA	NA	C228T	4-2	FULL	Yes	4.2	Yes	25.5	Int
35	G	P	48	F	H&N	30	0	No	+	3	9	0.5 (N)	NA	6-5	FULL	Yes	4.2	No	8.8	Low
36	G	R	58	M	H&N	27	2	No	+	1	7.5	2 (N/C)	NA	6-16	TAD	Yes	4.5	No	148.9	Low
37	G	R	87	F	PP	125	4	Yes	+	1	2	3 (N)	C228T	4-2	FULL	Yes	4.9	Yes	11.6	High
38	G	P*	51	F	Ext	95	32	No	+	9	1.5	0.5 (N)	C228T#	6-16	TAD	Yes	5.3	Yes	11.5	Low
39	G	R	64	M	PP	NA	3	No	+	NA	NA	NA	C228T	4-2	FULL	Yes	5.3	Yes	13.5	NA
40	G	R	32	M	Abd	NA	3	No	+	0	4.5	1.5 (N)	NA	6-16	TAD	Yes	6.0	Yes	92.8	NA
41	G	R	76	F	Ext	57	0	No	+	NA	NA	NA	NA	2-2	FULL	Yes	7.8	No	14.8	Low
42	G	P	55	M	Ext	90	0	No	+	NA	9	NA	NA	6-16	TAD	Yes	8.4	Yes	14.1	Low
43	G	R	53	F	Trunk	80	0	No	+	NA	NA	NA	NA	6-16	TAD	Yes	10.2	No	28.9	Low
44	G	R	76	M	Vis	130	0	No	+	NA	NA	NA	NA	7-4	FULL	Yes	10.4	Yes	28.6	Low
45	G	R	79	F	Ext	85	3	Yes	+	3	6	1 (N)	NA	6-16	TAD	Yes	10.7	No	36.5	Int
46	G	P	76	M	PP	210	0	No	+	NA	NA	NA	NA	4-2	FULL	Yes	11.1	Yes	11.1	Int
47	G	R	35	M	H&N	NA	8	No	+	0	6	0	NA	6-16	TAD	Yes	12.2	No	183.9	NA
48	G	R	43	F	Vis	NA	7	No	+	7.5	1.5	2 (N)	wt	4-2	FULL	Yes	13.7	No	17.6	NA
49	G	R	72	M	Trunk	40	13	No	+	9	9	1 (N)	NA	5-16	TAD	Yes	18.0	Yes	24.1	Low
50	G	R	69	M	Men	NA	5	No	+	2.5	5	3 (N)	C228T	4-2	FULL	Yes	20.8	No	20.8	CNS-G III
51	G	R	74	M	Ext	NA	6	No	+	3	9	1 (N/C)	NA	2-2	FULL	Yes	25.3	No	36.2	NA
52	G	R	54	F	PP	15	0	No	+	NA	NA	NA	NA	6-16	TAD	Yes	30.6	No	114.7	Low
53	G	R	50	M	H&N	60	0	No	+	NA	NA	NA	NA	6-16	TAD	Yes	32.5	No	48.5	Low
54	G	R	74	M	PP	NA	4	No	+	3	1	1 (N)	NA	4-2	FULL	Yes	33.4	No	57.1	NA
55	G	R	47	F	Abd	75	9	No	+	NA	NA	NA	wt	6-16	TAD	Yes	33.8	No	48.6	Low
56	G	R	74	F	Ext	90	6	Yes	+	2.5	3	2.5 (N)	NA	6int-2	FULL	Yes	37.6	No	64.9	Int
57	G	R	78	F	PP	100	8	No	+	2	0	0	NA	4-2	FULL	Yes	38.8	No	85.7	Int
58	G	R	52	F	PP	NA	8	No	+	1.5	3	1.5 (N)	NA	6-16	TAD	Yes	46.7	No	112.6	NA
59	G	R	50	F	Vis	130	0	No	+	NA	NA	NA	NA	6-2	FULL	Yes	51.2	No	76.3	Low
60	G	R	31	F	H&N	37	0	No	+	0	1.5	0	C228T	4-2	FULL	Yes	56.0	No	102.7	Low
61	G	R	34	M	Abd	190	4	No	+	1	1	0.5 (N)	NA	6-16	TAD	Yes	56.9	No	92.2	Int
62	G	R	66	M	PP	140	11	Yes	+	1	1	2 (N)	NA	4-2	FULL	Yes	57.8	Yes	106.0	High
63	G	R	50	F	Abd	30	14	No	+	7.5	3	1.5 (N)	NA	2-5	FULL	Yes	57.8	No	179.7	Low
64	G	R	20	M	Men	NA	7	Yes	+	2.5	4.5	0	wt	6-16	TAD	Yes	61.4	No	193.2	CNS-G III
65	G	R	49	M	H&N	85	3	No	+	NA	NA	NA	NA	2-1int	FULL	Yes	64.4	No	75.5	Low
66	G	R	66	F	Abd	NA	0	No	+	NA	NA	NA	NA	4-2	FULL	Yes	69.4	Yes	81.0	NA
67	G	R	52	M	Men	NA	3	No	+	1	9	1 (C)	NA	6-16	TAD	Yes	70.4	Yes	111.9	CNS-G II

68	G	R	37	F	Abd	100	0	No	+	NA	NA	NA	NA	2-1int	FULL	Yes	74.5	No	95.3	Low
69	G	R	61	F	PP	70	1	No	+	NA	NA	NA	NA	6-16	TAD	Yes	75.3	Yes	91.9	Low
70	G	R	42	F	Men	50	12	No	+	6	9	0.5 (N)	NA	6-16	TAD	Yes	75.8	Yes	80.8	CNS-G III
71	G	R	60	M	Men	NA	0	No	+	NA	NA	NA	NA	6-16	TAD	Yes	76.0	No	85.3	NA
72	G	R	27	F	Men	NA	6	No	+	4	9	1 (N/C)	NA	6-16	TAD	Yes	91.9	No	166.9	CNS-G III
73	G	R	33	F	Men	39	3	No	+	1.5	6	1 (N)	NA	6-16	TAD	Yes	92.5	No	138.0	CNS-G II
74	G	R	22	F	Men	NA	9	No	+	NA	NA	NA	NA	6-16	TAD	Yes	96.5	No	127.0	CNS-G III
75	G	R	40	F	Trunk	60	1	Yes	+	0	6	0	NA	5int-16	TAD	Yes	101.3	No	117.4	Low
76	G	R	56	F	PP	90	1	No	+	NA	NA	NA	NA	4-2	FULL	Yes	133.5	No	238.7	Low
77	G	R	52	F	PP	155	10	Yes	+	0.5	1	2.5 (N)	NA	4-2	FULL	Yes	138.5	No	139.2	High
78	G	R	28	F	Men	NA	1	No	+	0	5	0.5 (N)	NA	6-17	TAD	Yes	150.0	No	274.9	CNS-G I
79	G	R	67	M	Trunk	270	8	No	+	2.5	4.5	1 (N)	wt	4-2	FULL	Yes	157.8	No	191.5	High
80	G	R	59	F	Abd	75	6	Yes	+	1.5	4	2 (C)	NA	4-2	FULL	Yes	179.2	Yes	210.9	Int
81	G	R	59	M	H&N	40	3	No	+	NA	NA	NA	NA	6-17	TAD	Yes	197.2	No	233.8	Low
82	G	R	52	F	Abd	200	2	No	+	0.5	2.5	1.5 (N)	NA	6-17	TAD	Yes	229.4	Yes	325.8	Int
83	G	R	73	M	PP	NA	48	NA	+	NA	NA	NA	NA	2-2	FULL	NA	NA	NA	NA	NA

Abbreviations: Cohort: (L) Local; (G) GEIS; Specimen type: (P) Primary; (R) Recidive; Gender: (M) Male; (F) Female; Location: (PP) Pleuro-pulmonary; (Ext) Extremity; (Abd) Abdominal; (H&N) Head and Neck; (Vis); Visceral; (Men) Meningeal; pan-TRK IHC: (N) Nuclear staining; (C) Cytoplasmic staining; *TERT* promoter (*pTERT*) status: (wt) wild-type; (C228T or C228A) mutated at C228; (DFS) disease-free survival; (OS) overall survival, both expressed in months. Non-meningeal SFTs are stratified by m*Demicco* risk stratification system as Low, Intermediate (Int) or High; and meningeal SFTs are stratified by WHO Classification - CNS Tumours as I, II or III. (NA) Not available data. *Deddiferentiated SFTs. #*pTERT* homozygous mutation.
