# Supplementary material for: Evaluation of NAB2-STAT6 Fusion Variants and Other Molecular Alterations as Prognostic Biomarkers in a Case Series of 83 Solitary Fibrous Tumors
Source: Cancers (Basel). 2021 Oct 19;13(20):5237. doi: 10.3390/cancers13205237 (PMC8534228; doi:10.3390/cancers13205237)
Supplement: Supplementary file 1 [file cancers-13-05237-s001.zip › Table S2.pdf]

**Table S2.** Antibodies, suppliers, dilutions and scoring

| Marker | Antibody | Supplier | Dilution | Cut-off |
|---|---|---|---|---|
| P53 | CONFIRM anti-p53 (DO-7), mouse monoclonal antibody ($IgG_1$, kappa) | Roche | pre-diluted | Low: 0-1.5<br>High: 2-9 |
| NTRK | Anti-Pan Trk (ab181560), rabbit monoclonal | Abcam | 1/250 | Negative: 0<br>Positive: 0.5-9 |
| BCOR | Anti-BCOR (C-10 ) | Santa Cruz Biotechnology | 1/70 | Low: 0-5<br>High: 5.5-9 |
