# Supplementary material for: Evaluation of NAB2-STAT6 Fusion Variants and Other Molecular Alterations as Prognostic Biomarkers in a Case Series of 83 Solitary Fibrous Tumors
Source: Cancers (Basel). 2021 Oct 19;13(20):5237. doi: 10.3390/cancers13205237 (PMC8534228; doi:10.3390/cancers13205237)
Supplement: Supplementary file 1 [file cancers-13-05237-s001.zip › Table S3.pdf]

Table S3. Associations of NAB2-STAT6 fusion variants with clinicopathological parameters

	**Whole series**				**GEIS cohort**				**Local cohort**			
**NAB2-STAT6 fusion variant**	**6-16/17**	**4-2**	**other**	***p* value**	**6-16/17**	**4-2**	**other**	***p* value**	**6-16/17**	**4-2**	**other**	***p* value**
**Relative frequency**												
No. (%)	34 (41)	25 (30.1)	24 (28.9)		23 (46)	15 (30)	12 (24)		11 (33.3)	10 (30.3)	12 (36.4)	
**Sex**				0.617				0.964				0.471
Male	15 (42.9)	12 (34.3)	8 (22.9)		10 (45.5)	7 (31.8)	5 (22.7)		5 (38.5)	5 (38.5)	3 (23.1)	
Female	18 (39.1)	13 (28.3)	15 (32.6)		13 (46.4)	8 (28.6)	7 (25)		5 (27.8)	5 (27.8)	8 (44.4)	
**Age (years)**				**0.003**				**0.004**				0.603
Mean ± SD	47.9 ±14.3	61.6 ±14.5	58.2 ±14.0		459 8± 14.6	63.38±14.12	59.91±15.48		52.4±13.2	58.6 ± 15.5	56.3±12.7	
Median (Range)	51 (20-79)	65 (31-87)	56 (37-79)		51 (20-79)	66 (31-87)	61 (37-76)		50 (27-70)	65 (32-77)	56 (38-79)	
**Size (mm)**				**0.023**				**0.027**				0.438
Means ± SD	83.4 ± 53.0	126.1 ± 62.6	77.2 ± 44.9		79.7 ± 54.5	143.6 ± 68.4	75.2 ± 37.8		93.6 ± 53.1	103.6 ± 49.9	79.5 ±5 4.1	
Median (Range)	75 (15-200)	110 (37-270)	70 (30-210)		73 (15-200)	140 (37-270)	73 (30-130)		120 (15-140)	98 (37-200)	70 (35-210)	
**Location**				**> 0.001**				**0.010**				**0.007**
Abdominal/ Visceral	11 (45.8)	5 (20.8)	8 (33.3)		4 (36.4)	3 (27.3)	4 (36.4)		7 (53.8)	2 (15.4)	4 (30.8)	
Pleuro-pulmonary	4 (19)	15 (71.4)	2 (9.5)		3 (23.1)	9 (69.2)	1 (7.7)		1 (12.5)	6 (75)	1 (12.5)	
Meningeal	10 (90.9)	1 (9.1)	0 (0)		8 (88.9)	1 (11.1)	0 (0)		2 (100)	0 (0)	0 (0)	
Extremity	3 (30)	1 (10)	6 (60)		3 (50)	0 (0)	3 (50)		0 (0)	1 (25)	3 (75)	
Head and Neck	4 (44.4)	1 (11)	4 (44.4)		4 (57.1)	1 (14.3)	2 (28.6)		0 (0)	0 (0)	2 (100)	
Trunk	1 (16.7)	1 (16.7)	4 (66.7)		1 (25)	1 (25)	2 (50)		0 (0)	0 (0)	2 (100)	
**Necrosis**				0.137				0.336				0.317
Absent	27 (44.3)	16 (26.2)	18 (29.5)		21 (51.2)	11 (26.8)	9 (22)		6 (30)	5 (25)	9 (45)	
Present	3 (23.1)	7 (53.8)	3 (23.1)		2 (25)	4 (50)	2 (25)		1 (20)	3 (60)	1 (20)	
**Mitotic count (10 HPFs)**				0.06				0.425				**0.008**
<4 (%)	18 (40)	10 (22.2)	17 (37.8)		14 (51.9)	6 (22.2)	7 (25.9)		4 (22.2)	4 (22.2)	10 (55.6)	
≥4 (%)	16 (47.1)	13 (38.2)	5 (14.7)		9 (39.2)	9 (39.1)	5 (21.7)		7 (63.6)	4 (36.4)	0 (0)	
**Recurrence/progression**				**0.031**				-				0.078
No	4 (19)	7 (33.3)	10 (47.6)						4 (19)	7 (33)	10 (47.6)	
Yes	27 (49.1)	16 (29.1)	12 (21.8)						4 (66.7)	1 (16.7)	1 (16.7)	
**Outcome**				0.191				0.295				0.291
Alive	23 (39)	16 (27.1)	20 (33.9)		16 (48.5)	8 (24.2)	9 (27.3)		7 (26.9)	8 (30.8)	11 (42.3)	
Dead of disease	8 (47.1)	7 (41.2)	2 (11.8)		7 (43.8)	7 (43.8)	2 (12.5)		1 (100)	0 (0)	0 (0)	

Data are given as number (%) unless otherwise indicated.
Significant P values are indicated in bold.
